# Data in support of environmental controls on the characteristics of mean number of forest fires and mean forest area burned (1987–2007) in China

**DOI:** 10.1016/j.dib.2015.07.025

**Published:** 2015-07-29

**Authors:** Yu Chang, Zhiliang Zhu, Rencang Bu, Yuehui Li, Yuanman Hu

**Affiliations:** a State Key Laboratory of Forest and Soil Ecology, Institute of Applied Ecology, Chinese Academy of Sciences, Shenyang 110164, China; b U.S. Geological Survey, Reston, VA 20192, USA

## Abstract

Fire frequency and size are two important parameters describing fire characteristics. Exploring the spatial variation of fire characteristics and understanding the environmental controls are indispensable to fire prediction and sustainable forest landscape management. To illustrate the spatial variation of forest fire characteristics over China and to quantitatively determine the relative contribution of each of the environmental controls to this variation, forest fire characteristic data (mean number of forest fires and mean burned forest area) and environmental data (climate, land use, vegetation type and topography) at provincial level were derived. These data sets can potentially serve as a foundation for future studies relating to fire risk assessment, carbon emission by forest fires, and the impact of climate change on fire characteristics. This data article contains data related to the research article entitled “Environmental controls on the characteristics of mean number of forest fires and mean forest area burned (1987–2007) in China” by chang et al. [1].

Specifications table

**Table:** 

Subject area	Biology
More specific subject area	Forestry
Type of data	Table
How data was acquired	The data was produced by reanalyzing data from the following websites:
	http://www.cfsdc.org/
	http://cdc.nmic.cn/
	http://www.stats.gov.cn/
	http://westdc.westgis.ac.cn/
Data format	Analyzed
Experimental factors	NA
Experimental features	NA
Data source location	Shenyang, PR China
Data accessibility	Analyzed data sets are directly provided with this article

Value of the data

● The data can be used to analyze dynamic changes of forest fire characteristics.
● The data provides basic information needed for national level forest fire management planning.
● The data can potentially serve as a foundation for future studies relating to fire risk assessment, carbon emission by forest fires, and the impact of climate change on fire characteristics.

## 1 Data, experimental design, materials and methods

### 1.1 Calculation of forest fire characteristics

We quantified two parameters of fire characteristics including fire size and fire frequency. We calculated the mean burned forest area from 1987 to 2007 for each province as a proxy of fire size and the mean number of fires as a proxy of fire frequency based on forest fire statistical data (http://www.cfsdc.org/).

### 1.2 Derivation of environmental data

#### 1.2.1 Climatic variables

We calculated the monthly de Martonne aridity index [2] values according to Eq. (1) based on monthly climate data (http://cdc.nmic.cn/) and averaged to obtain an annual mean de Martonne aridity index for each year respectively. The Kira arid index [3] was also calculated for each year according to Eqs. (2) and (3). Thus the average annual mean de Martonne aridity index and Kira arid index was derived by averaging their values across 21 years. We finally calculated the average annual mean temperature, precipitation, de Martonne aridity index and Kira arid index for each province with the support of ArcGis 9.3 software.

(1) $$dM=\frac{P_{monthly}}{T_{Monthly}+10}$$

Where *dM* is the de Martonne aridity index, *P*_*monthly*_ is the monthly precipitation in mm, *T*_*monthly*_ is the monthly mean temperature in °C.

(2) $$WI=\sum_{i=1}^{n}\left(T_{monthly}-5\right)$$

(3) $$kira=\{\begin{array}{cc} P/(WI+20) & \mathrm{when}\\ 2P/(WI+140) & \mathrm{when} \end{array}\begin{array}{c} WI=0-100\\ WI>100 \end{array}$$

Where *WI* is the warm index, the sum of monthly temperature greater than 5 °C, *n*=1,2…12, *Kira* is the Kira arid index, *P* is annual precipitation in mm.

#### 1.2.2 Land use types

We calculated the proportion of forest land, grassland, farmland and built-up land for each province based on the land use map of China (http://westdc.westgis.ac.cn/). The percentage of forest land and grassland could act as a proxy for fuel type which is a key factor affecting fire characteristics. The percentage of farmland and built-up land could reflect the degree to which human utilized the land resources.

#### 1.2.3 Forest vegetation types

We calculated the percentage of coniferous forests, mixed forests and deciduous forests in each province respectively to represent the variations of forest vegetation based on vegetation map of China (http://westdc.westgis.ac.cn/).

#### 1.2.4 Human influence factor

Population density is a factor commonly used when studying fire characteristis. We collected a digital administrative map of China with county and city boundaries (1:250,000), assigned the population census data of each city and county (http://www.stats.gov.cn/) to their corresponding polygons using ArcGis 9.3, calculated the population density and transformed the vector map to raster format with 1 km spatial resolution and finally calculated the average population density for each province.

#### 1.2.5 Topographic factor

Topography is a critical factor influencing fire characteristics. We computed the standard deviation of altitude for each province to represent topographic complexity based on the DEM with 1 km resolution (http://westdc.westgis.ac.cn).
